# Assessing Dietary Intakes from Household Budget Survey in Armenia, 2008–2019

**DOI:** 10.3390/foods11182847

**Published:** 2022-09-14

**Authors:** Seda Stepanyan, Davit Pipoyan, Meline Beglaryan, Nicolò Merendino

**Affiliations:** 1 Center for Ecological-Noosphere Studies of NAS RA, Abovyan 68, Yerevan 0025, Armenia; 2 Department of Ecological and Biological Sciences (DEB), Tuscia University, Largo dell’Università snc, 01100 Viterbo, Italy

**Keywords:** dietary intake, household budget survey, macronutrient

## Abstract

Household budget surveys are used regularly to estimate dietary intakes. The study aims to assess the trends in food consumption and nutrient intake, according to 14 dietary indicators from household budget surveys in Armenia. Data on food consumption was obtained from Armenian Integrated Living Conditions Surveys, 2008–2019. The results indicate that the consumption of all types of foods, including plant-origin has decreased, whereas the consumption of foods of animal origin has mostly stayed stable. Over time, the energy and macronutrient intakes of Armenians have decreased, while the contribution of each food group to total energy and nutrient intake has not changed. More than 50% of total energy, protein, and carbohydrate intake is attributable to cereals and bakery products. The population is characterized by macronutrient variations; the amounts of energy and carbohydrate intake are below the recommended values set by WHO/FAO, total fat intake is at the highest recommended level, while the amount of protein exceeds the threshold. Based on the findings there is an urgent need to increase awareness of nutritional requirements and a need to change widespread dietary practices, such as irregular meal intake and omission of breakfast.

## 1. Introduction

Food security and nutrition are major global challenges [1]. Over the past decades, food consumption patterns and food availability have changed drastically mainly due to alterations in food habits, changing lifestyles, and the introduction of new, healthier, or processed foods. This, in turn, has led to changes in overall energy and macronutrient intake, resulting in a deficiency or excess of these substances [2,3,4]. Macronutrients, including protein, carbohydrates, and fats play a vital role in ensuring a healthy diet and preventing many diseases. They each have a set of unique properties, and all are a source of energy [5].

To study changes in food consumption patterns, as well as food and macronutrient intakes, dietary surveys are used widely at the household or individual level [6,7]. These surveys serve as a basis for developing programs or policies to tackle nutrient gaps or the risks of inadequate or excess intakes. There are various methods available to collect dietary intake data and depending on the complexity and cost of dietary surveys, countries use different methods. Due to their low cost, Household Consumption and Expenditure Surveys (HCES) have become increasingly accessible and today are widely used in more than 120 countries [8,9,10].

In Armenia, household budget surveys are routinely conducted on nationally representative samples. The first household survey, titled Integrated Living Conditions Survey (ILCS) was conducted in 1996. Starting from 2001, it has been conducted every year. ILCS provides comprehensive information regarding the living conditions of the population of Armenia and a quantitative evaluation of its major indicators. The results of the survey are analyzed according to the following indicators: consumption, income, expenses, consumption of basic food, the existence of long-term goods, affordable health and educational systems, social transfers, etc. [11].

Several studies have been conducted to explore the nutritional status in Armenia and the cost of the diet. These studies mainly point out the fact that although people can achieve the daily recommended calorie intake, the nutrient requirements might not be met, leading to malnutrition and long-term health implications [12,13]. However, these studies do not focus on the macronutrient intake trends, or on the long-term changes in food consumption expenditures due to price volatility. Therefore, there is a need to investigate dietary habits among Armenian households, especially on an individual level to get more accurate data based on age, gender, education, or income. It is important to note that, currently, there is an absence of an official individual-based methodology for investigating dietary habits among Armenian households, which makes it difficult for policymakers to monitor changes using evidence-based approaches. Several studies have been conducted by the Informational-Analytical Center for Risk Assessment of Food Chain, utilizing individual-based surveys [14,15]. Considering the very limited data available on the dietary intake of the Armenian population, the purpose of the present study is to explore food consumption trends and assess major macronutrient intakes from 2008–2019, using household budget surveys. Moreover, this study aims to describe the changes in food consumption expenditures over the same period based on household income and Consumer Price Index (CPI).

## 2. Materials and Methods

### 2.1. Dietary Survey

To assess dietary intake, the latest available data ranging from 2008 to 2019 were obtained from ILCS. Nationally representative samples of 32,756 and 18,496 individuals took part in the ILCS conducted by the National Statistical Service of the Republic of Armenia in 2008 and 2019, respectively. A total of 7872 and 5184 households completed the survey between January and December of 2008 and 2019, respectively. Households recorded over one month all their consumption and expenditures on food, non-food products, and services (including details, such as name, quantity, cost, and place of purchase of the product). Other data collected included information on household composition and living conditions, employment status, educational level, health condition, and other information [16].

In this study, quantities for food consumption and the estimated energy and nutrient content of foods were analyzed on an “as consumed” basis. Estimates of human energy and nutrient intake were assessed based on the food composition table developed jointly by the National Statistical Service of the Republic of Armenia, the Ministry of Agriculture, and the Food and Agricultural Organization (FAO) of the United Nations. The food composition table was compiled according to INFOODS standards and food component identifiers [17].

### 2.2. Dietary Intake Assessment

Dietary indicators were chosen to capture an individual’s intake from main sources. Overall, 14 dietary indicators, including 10 food groups (cereals and bakery products, meat and meat products, fish, milk and milk products and eggs, fats and oils, fruits, vegetables, potato, sugar, honey and confectionery, mineral water (bottled) and juices), 3 macronutrients (protein, carbohydrate, total fats), and total energy were included in the study. Each food group consisted of food products that have a consumption of more than 1 g/day (Table 1).

Every item in the survey food list was matched to an entry in a food composition table to identify each food’s nutrient content per 100 g. The intake of each nutrient was then calculated by multiplying the weight of each consumed food by the concentration of the nutrient in that food using the following formula [10,18]:

(1) $$I=\sum \left(W_{1}\times C_{1}+W_{2}\times C_{2}+W_{3}\times C_{3}+\ldots W_{n}\times C_{n}\right),$$

where I is the intake of the nutrient, $W_{1}$ is the weight of the first food product (g/day), $C_{1}$ is the concentration of the nutrient in the first food product (g), and n is the number of food products.

Metabolizable energy values have been calculated from the amount of protein, fat, and carbohydrates in the foods, applying the energy conversion factors.

### 2.3. Food Consumption Trends and Statistical Analysis of Data

Pearson’s correlation was used to assess the correlation between average monthly per capita household income, CPI, and food consumption expenditure from 2008–2019. A bivariate linear regression has been conducted to investigate how much CPI predicts consumption expenditures on food products. All analyses were conducted at the 0.05 and 0.01 significance levels. Statistical analysis of data was performed using Python. Three main Python packages were applied: Scikit-learn, NumPy, and Matplotlib.

## 3. Results and Discussion

### 3.1. Estimates of Food Availability

The food consumption data obtained from the ILCS survey reflects the food acquired by (available to) the household during the reference period. Data on food consumption between 2008 and 2019 for per capita households are presented in Table 2. During this period, the total monthly food consumption decreased by 7.25%, from 43.56 kg to 40.40 kg.

#### 3.1.1. Cereals and Bakery Products

Overall, there was a decreasing trend in cereals and bakery product consumption between 2008 and 2019 (20% reduction). High-grade white bread and lavash were the subgroups that contributed the most to cereals and bakery product intake. Consumption of sweet cereal products (biscuits, pastries, cakes) went down substantially. Similarly, the intake of rice and pasta also decreased compared to previous years.

#### 3.1.2. Meat and Meat Products, Fish

Total meat and fish consumption stayed mostly stable over the years with slight fluctuations. Chicken, beef, and veal were the subgroups that contributed the most to meat consumption.

#### 3.1.3. Milk and Milk Products

Overall, the consumption of milk and milk products decreased very slightly (approximately 11%) except for chicken eggs. The latter was characterized by a small increase in consumption amount (approximately 20%). Matsun and cheese had the largest share in milk product intake.

#### 3.1.4. Fats and Oils

Over the years, the consumption of butter and plant-based oil decreased by as much as 75% and that of vegetable oil stayed mostly stable. Plant-based oils have the largest share in fats and oils intake.

#### 3.1.5. Fruits

There was a reduction in the average consumption of fruits from 2009 to 2019; the amount decreased by 20%, from 4.5 kg to 3.6. Watermelon, apple, grapes, and apricot were the subgroups with the largest share of the total intake.

#### 3.1.6. Vegetables

Vegetable consumption (excluding potatoes) decreased in 2010 and 2019 and stayed stable throughout the rest of the years. The average monthly vegetable consumption ranged from 5.9 kg to 6.5 kg. Similarly, potato intake decreased by almost 18%, amounting to 3.2 kg per month in 2019.

#### 3.1.7. Sugar, Honey, and Confectionery

The average weight of total sugar, honey, and confectionery consumption decreased twice in 2019 compared to 2008. Granulated sugar and fruit compotes were the subgroups with the largest share in the intake amount.

**Figure 1:**
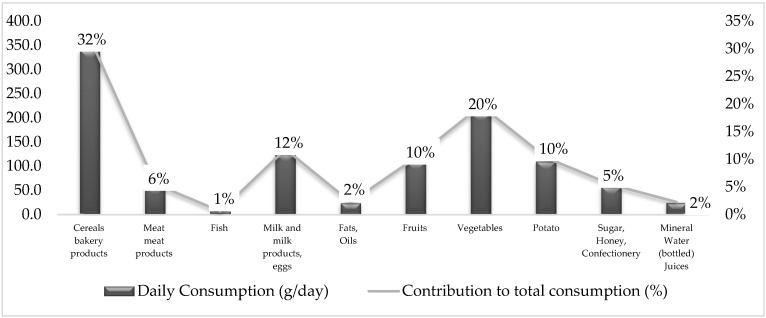
Daily consumption of main food groups per capita (g/day) and structure of consumed food in the Integrated Living Conditions Surveys. Armenia 2019.

Figure 1 shows the estimated food consumption for 10 food groups based on 2019 ILCS data. The diet is characterized mainly by cereals, vegetables, milk and milk products, and eggs (Figure 1).

It should be noted that according to FAO recommendations, the intake of fruits and vegetables (excluding tubers) should be 400 g/day or more [19]. Based on this study, the consumption of fruits and vegetables (excluding potatoes) approximately equals 320 g/day which is less than the recommended amount. The difference can be attributed to the fact that fruits and vegetables consumed away from home are not accounted for in ILCS. Moreover, other factors, including price, food availability, cultural conditions, and nutritional knowledge can also contribute to the low consumption of these food items.

### 3.2. Energy Intake and Energy Contributions of Macronutrients

**Figure 2:**
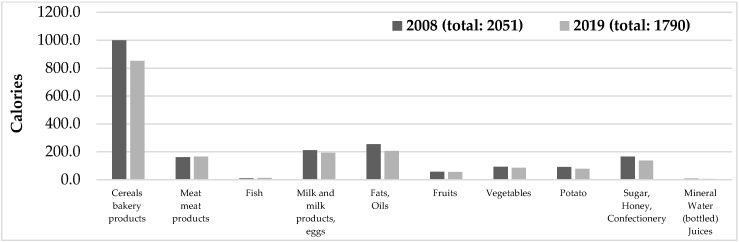
Average daily per capita calories (kcal/day). Armenia. 2008 and 2019.

The amount of total energy estimated from the consumption of all 10 food groups amounted to 2051 and 1790 kcal/day per capita in 2008 and 2019, respectively (Figure 2).

In 2008, the estimates of protein, carbohydrates, and total fats were equal to 83.15 g/day, 265.55 g/day, and 69.71 g/day, respectively. Meanwhile, in 2019, the estimates of protein, carbohydrates, and total fats were equal to 74.42 g/day, 226.56 g/day, and 62.73 g/day, respectively. Armenia is a member of the Eurasian Economic Union (EEAU) and follows its Customs Union Technical regulations for food product labeling [20]. The latter defines uniform requirements for labeling food products including the average daily need for basic nutrient substances and energy. According to the regulation, the required daily intake of energy, protein, carbohydrates, and total fats should be 2500 kcal, 75 g, 365 g, and 83 g, respectively. Except for protein, the amount of energy, carbohydrates, and total fat intakes estimated both in 2008 and 2019 are below the recommended values. According to the World Health Organization and FAO (WHO/FAO), the average energy requirement values range from 1650–3150 kcal/day for women and 2100–3600 kcal/day for men depending on physical activity level. Thus, the amounts of energy estimated in 2008 and 2019 are below the requirement [21]. This difference may be because ILCS does not account for mixed dishes (e.g., spas, harissa, meatball soup, etc.), foods consumed (acquired) away from home (e.g., fast food), food waste, storage food, etc. In 2020, the Informational-Analytical Center for Risk Assessment of Food Chain of Center for Ecological Noosphere Studies of RA investigated fast food consumption habits among the Yerevan population via a food frequency questionnaire (FFQ), based on which, fast-food intake ranged from 14.68 to 76.09 g/day per person. Therefore, considering the number of nutrients present in fast food, this group also adds up to the estimates of total energy and macronutrient intake of the current study.

#### Food Sources of Dietary Energy

**Figure 3:**
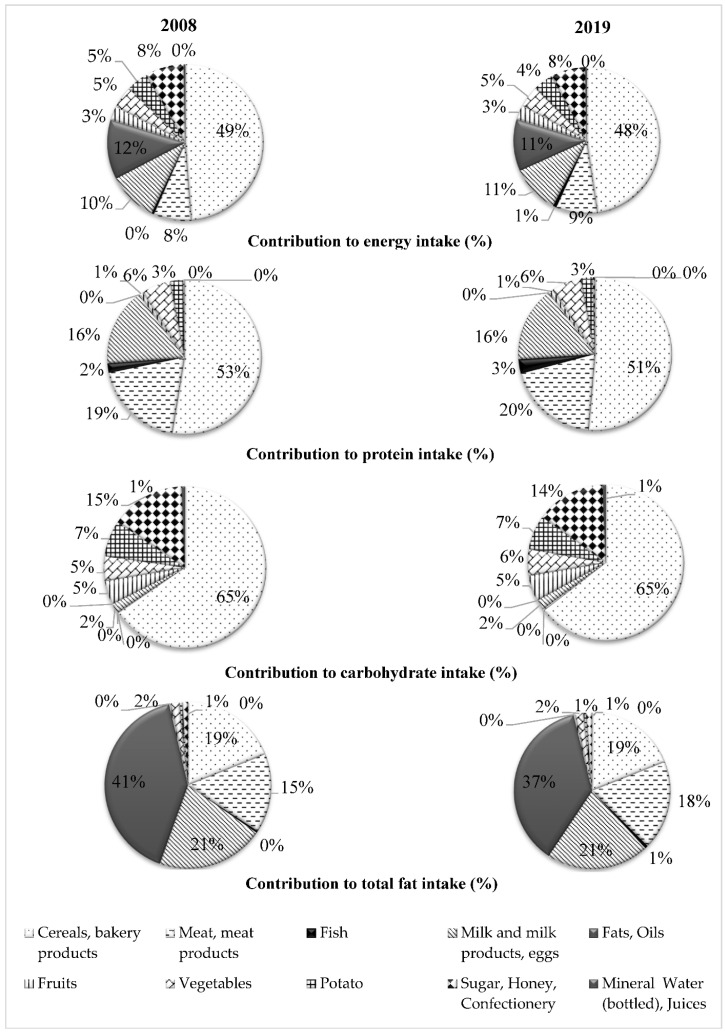
Contribution of food groups to energy and macronutrient intake (%). Armenia. 2008 and 2019.

Figure 3 depicts the percent contribution of each food group to daily intakes of energy, protein, carbohydrates, and total fats in 2008 and 2019. There are no significant changes in terms of the contribution of food groups to energy and macronutrient intakes between 2008 and 2019. Cereals and bakery products contribute the greatest proportion of all studied macronutrients (except for total fats) and total energy. Milk and milk products, vegetables, and potatoes make up 12%, 5%, and 4% of total energy, respectively. Protein intake is characterized mainly by cereals and bakery products, followed by meat and meat products and milk and milk products. Carbohydrate intake is attributable mostly to cereals and bakery products, potatoes, as well as sugar, honey, and confectionery. Lastly, total fats are received mainly from fats and oils, milk and milk products, and eggs.

### 3.3. Contribution of Food Groups to Energy and Macronutrient Intakes in Different Countries

**Figure 4:**
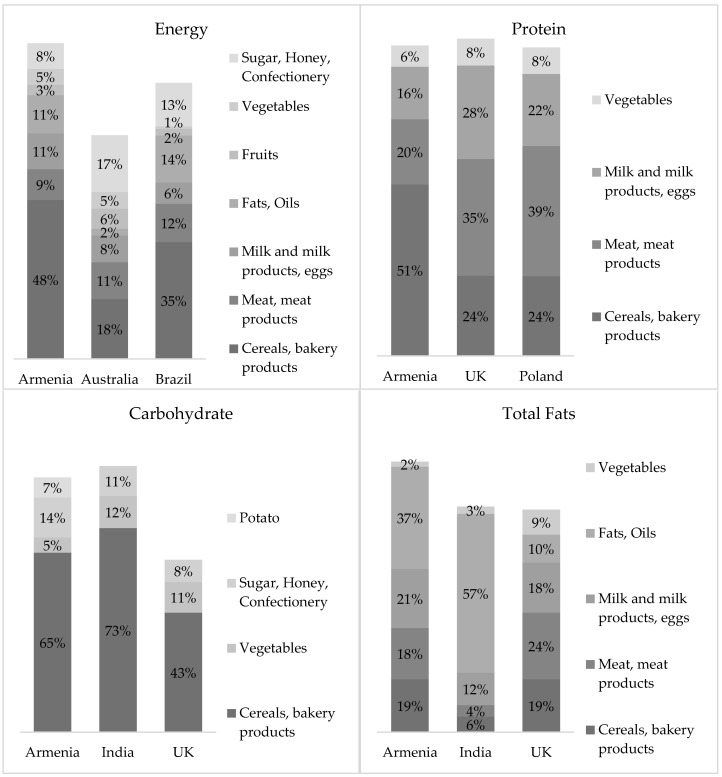
Contribution of food groups to total daily energy and macronutrient intakes in different countries (%).

Figure 4 compares the contribution of food groups to energy and macronutrient intakes between Armenia and other countries (using the foods-as-consumed approach for Australia and the UK, Poland, India, and the foods-as-purchased approach for Brazil) [22,23,24,25,26].

#### 3.3.1. Total Energy Intake

Compared to Australian and Brazilian diets, in the Armenian diet, cereals and bakery products have a very large contribution to total daily energy intake, which may be because this food group has a large share of the total diet.

#### 3.3.2. Protein Intake

Regarding protein intake, a similar pattern was observed between UK and Polish diets with meat and meat products having the largest contribution. However, in the Armenian diet, the highest contribution to protein intake was attributable to cereals and bakery products.

#### 3.3.3. Carbohydrate Intake

Cereal and cereal products were the main source of carbohydrates for adults in all countries. The Armenian estimate of this food group was in between Indian and UK estimates. The rest of the food groups had an approximately equal contribution to carbohydrate intake in the studied countries.

#### 3.3.4. Total Fats Intake

The contribution of food groups to total fat intake differed between the studied countries. In Armenia, it was characterized mainly by fat and oil product intake, in India by milk and milk products, and in the UK by meat and meat products.

In this study, the estimated metabolizable energy values of protein, carbohydrates, and total fats range from 297.67–332.6, 906.22–1062.22, and 564.57–627.39 kcal/day, respectively. This implies that in the Armenian diet 50–52% of total energy is received from carbohydrates. According to FAO, an optimum diet should consist of at least 55% of total energy coming from carbohydrates obtained from a variety of food sources [19]. Therefore, the energy value received from carbohydrates is almost consistent with the required level set by FAO. This amount also falls within the reference intake range of 45–60% set by EFSA [27]. The estimated daily protein intake (74.42–83.15 g/day) makes 16–17% of total energy. When considering a mean body weight of 75 kg, daily protein intake ranges from 0.992 to 1.108 g/kg bw per day, which is slightly higher than the population reference intake of 0.83 g/kg bw per day set by EFSA and the requirement of 0.75 g/kg bw per day set by WHO/FAO [28,29]. Total fat makes up 30–31% of total energy; this value is at the upper level of the reference intake range (20–35%) set by EFSA and at the highest required level of total energy (30–35%) set by WHO/FAO [30,31].

### 3.4. The Impact of Income and CPI Changes on Food Consumption Expenditure, 2008–2019

**Figure 5:**
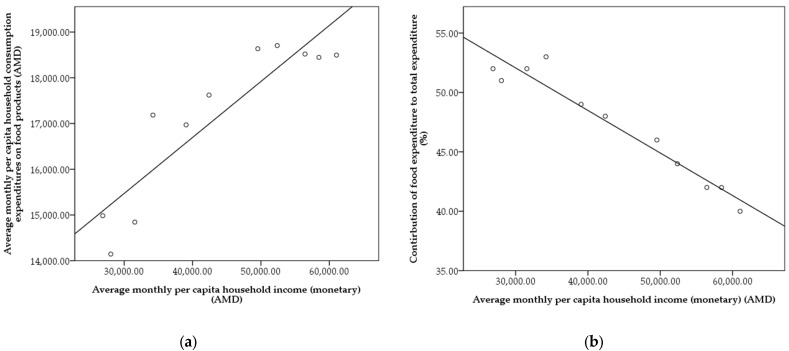
Relationship between per capita household income and food expenditure (**a**), and relationship between per capita household income the share of food consumption expenditure to total expenditure (**b**). Armenia. 2008–2019.

According to ILCS, the average per capita household income has increased from 26,866 Armenian Dram (AMD) to 61,047 AMD from 2008 to 2019. Similarly, the average monthly per capita household consumption expenditures on food products have increased from 14,984 AMD to 18,496 AMD in the same period [32]. Figure 5a illustrates the relationship between income and consumption expenditure on food products. There is a strong positive correlation (r = 0.912; *p* = 0.000) between household income and food expenditure. However, throughout the years as household income has increased, the share of food expenditures to total expenditures has decreased from 52% to 41% due to an increase in CPI. According to Figure 5b, there is a strong negative correlation (r = −0.973; *p* = 0.000) between household income and the share of food expenditure to total expenditure. According to the National Statistical Committee of Armenia, during 2008–2020, CPI increased from 137.6 to 201.3 over the base year of 2000 [33].

To investigate how much the consumer price index predicts the share of food consumption expenditure, a bivariate linear regression analysis was performed. Linear regression assumptions of normal distribution were checked by Kolmogorov-Smirnov and Shapiro–Wilk tests, as well as skewness and kurtosis. Since the variables were normally distributed, there was no need for data transformation. Multicollinearity and correlations between each variable were checked using VIF and tolerance test. For each variable, tolerance values were greater than 0.2, and values of VIF were less than 3.

The consumer price index negatively and significantly (β = −0.200, *p* = 0.000) affected the share of food consumption expenditure at the bivariate level. Overall, the regression model was significant (F = 37.993, *p* = 0.000) and had an adjusted R^2^ of 0.771. This indicates that the consumer price index explains 77% of the variance in the share of food consumption expenditure.

When investigating the correlation between each food type and CPI, it can be concluded that there were many statistically significant associations between individual food consumption and CPI from 2008–2019. There has been a significant decrease in the average monthly consumption of basic food products (bread and bread products, potatoes, fruits and berries, cheese, butter and oil, and sugar) due to an increase in CPI (Table 3). It has been revealed that the average monthly consumption of basic food products has decreased despite the increase in household income.

According to the Statistical Committee of Armenia, the CPI continued to increase during the first quarter of 2021, amounting to 211.76 (over the base year of 2000). Inflation has been recorded in 12 food commodity groups. Compared to the same period of the previous year, the price of sugar increased by 48.6%, fats and oils by 20.4%, milk, cheese, and eggs by 12.3%, fruits by 10.4%, non-alcoholic drinks by 10.2%, bread and cereals by 8.9%, vegetables by 8.0%, coffee, tea, cocoa by 2.3%, meat by 1.6%, fish and seafood by 0.6%, and confectionery by 0.4%. Moreover, during the observed period, the price of imported wheat increased by 29.7% compared to the same period of the previous year [34].

### 3.5. Meeting Dietary Recommendations

A study conducted in 2017 reports that while average Armenians can afford a healthy diet, their diet does not meet the dietary reference intake recommendations. This can be attributed to the fact that most Armenians lack nutrition knowledge, are inclined to culturally accepted diets, and are unaware of which foods are healthy and which ones are not. These knowledge gaps lead to diet-related health issues (for example, high blood pressure, high blood cholesterol, high blood glucose, and anemia) which are very widespread and concerning in Armenia [35]. Noncommunicable diseases (NCDs) account for an estimated 93% of all deaths in Armenia [36]. Another cross-sectional study conducted in Yerevan, Armenia, showed that the mean awareness regarding nutritional information was low (24.1%), particularly regarding relationships between diet and diseases. More than 25% of the respondents incorrectly believed that osteochondrosis was related to a high intake of salt [36]. 

In 2019, the World Food Programme carried out a food security and vulnerability assessment among Armenian households. According to the study, the dietary habits of the population in Armenia have deteriorated throughout the years, with the most vulnerable households sharply decreasing their intake of meat products, fruits, vegetables, and dairy products. Despite economic growth, levels of food insecure households almost doubled from 2008 to 2010 and remained at 15% in 2017. Unhealthy dietary habits are a norm in Armenia. The average Armenian consumes around two times more salt, four times more staple foods (i.e., baked goods and potatoes) than the recommended maximum intake level, and around two times fewer fruits and vegetables as compared to the recommended optimal intake [37]. To understand whether the current structure of food consumption in Armenian households corresponds to the principle of sustainable consumption, a comparison with the UK’s recommended “Eatwell Plate” has been made [38]. According to this, the ideal proportions of food types for a healthy diet are as follows: bread, rice, potato, pasta, and other starchy foods should make up 33% of the total diet, meanwhile, in Armenia, this number is at 42%. Fruits and vegetables should make up another 33% of the total diet, while in Armenia they make up 30%. The recommended share of milk and dairy products is 15%, but in Armenia, it is 12% (including eggs). The ideal proportion of meat, fish, eggs, and other non-dairy sources of protein is 12%, meanwhile, in the Armenian diet, it is only 7% (excluding eggs). Lastly, foods high in fat or sugar should make up 8% of the total diet, while in Armenia they make up almost as much (9%). Overall, there is a major difference in the share of starchy foods, fruits and vegetables, and animal-origin foods [38].

## 4. Conclusions

The results indicate that the consumption of most food products, including basic food products (bread and bread products, potatoes, fruits, cheese, butter, and oil) decreased from 2008–2019. Although there has been an increase in household income, the decrease in consumption amounts was attributable to an increase in CPI. The consumption of foods of animal origin has mostly stayed stable. Energy and macronutrient intakes of Armenians have decreased in 2019 compared to the average intakes obtained in previous ILCS surveys since 2008. The population is characterized by macronutrient variations; while the amounts of energy and carbohydrate intake are below the recommended values set by WHO/FAO and Customs Union Technical Regulation, total fat intake is at the highest recommended level set by WHO/FAO, and the amount of protein exceeds the threshold. The estimated amount of total energy is below the required level across the entire studied period. It is noteworthy that in the Armenian diet more than 50% of total energy, protein, and carbohydrate intake is attributable to cereals and bakery products, which is indicative of poor dietary diversity.

Based on the findings there is an urgent need to increase awareness of nutritional requirements and a need to change widespread dietary practices, such as irregular meal intake and omission of breakfast. This will be a small, but crucial step towards enabling nutritious choices for different population groups, and ensuring that every person has the minimum level of access to a diversified and nutritious diet.

A limitation of this study is that it excludes foods consumed out of home as well as foods acquired but not consumed by household members. Moreover, it does not estimate the distribution of usual nutrient intakes among household members. To reliably assess the prevalence of nutrient inadequacy (or excess) in a group of individuals, it is necessary to consider factors such as gender, age, and consumption of all types of food in that group. To obtain this information, it is recommended to conduct a 24-h recall and observed weighed food record surveys since they are regarded as the most precise and preferred sources of these data. Moreover, this can serve as a basis for formulating evidence-based data to support integrated policy decisions and a multi-level framework of action to better tackle nutritional issues.

## Figures and Tables

**Table 1:** Number of products in each food group.

Food Groups	Number of Products in Each Group
Cereals and bakery products	11
Meat and meat products	10
Fish	3
Milk and milk products, eggs	9
Fats and Oils	3
Fruits	14
Vegetables	22
Potato	1
Sugar, Honey, Confectionery	8
Mineral Water (bottled), Juices	7
**Total**	**88**

**Table 2:** Average Monthly Per Capita Consumption of Basic Food Products (kg/month). Armenia. 2008–2019.

Food Groups	Year											
	2008	2009	2010	2011	2012	2013	2014	2015	2016	2017	2018	2019
***Cereals and bakery products***	12.7	12.3	11.9	12.2	12.2	11.8	11.3	11.4	11.3	10.6	10.5	10.1
***Meat and meat products***	2	2	1.8	1.8	1.9	1.9	1.9	1.9	2	1.9	2	2.1
***Fish***	0.2	0.2	0.1	0.1	0.2	0.2	0.2	0.2	0.2	0.2	0.2	0.3
***Milk, matsun***	1.7	1.7	1.7	1.7	1.5	1.5	1.7	1.8	1.8	1.8	1.6	1.5
***Cheese***	1.06	1	0.8	0.8	0.9	0.8	0.8	0.9	0.9	0.8	0.8	0.8
***Eggs (in pieces)***	10.1	10.5	11.1	11.2	10.8	10.4	10.9	10.5	10.7	10.6	11.3	12
***Butter and oil***	0.4	0.4	0.3	0.2	0.2	0.3	0.2	0.2	0.2	0.2	0.1	0.1
***Vegetable oil and other oils***	0.6	0.5	0.5	0.6	0.4	0.4	0.5	0.5	0.6	0.6	0.6	0.5
***Fruits and berries***	3.9	4.5	4.3	4	4.2	3.9	3.8	3.9	3.9	3.5	3.6	3.6
***Vegetables***	6.3	6.1	5.7	6.5	6.6	6.6	6.4	6.6	6.6	6.2	6.5	5.9
***Potato***	3.9	3.9	3.9	3.6	3.6	3.5	3.4	3.5	3.4	3.3	3.3	3.2
***Sugar, honey, confectionery***	0.7	0.7	0.6	0.6	0.6	0.6	0.5	0.6	0.6	0.4	0.4	0.3

**Table 3:** Pearson Correlation between food consumption, income, and CPI. Armenia. 2008–2019.

Food Type	Average Monthly Per Capita Household Income (Monetary) *p* (Significance)	Consumer Price Index over the Base Year of 2000 *p* (Significance)
**Bread and bread products**	−0.935 ** (0.000)	−0.872 ** (0.001)
**Potato**	−0.949 ** (0.000)	−0.959 ** (0.000)
**Vegetables and vegetable crops**	0.190 (0.553)	0.295 (0.352)
**Fruits and berries**	−0.802 ** (0.002)	−0.749 ** (0.005)
**Meat and meat products**	0.382 (0.221)	0.206 (0.520)
**Milk and yogurt**	0.009 (0.977)	−0.074 (0.819)
**Cheese**	−0.534 (0.074)	−0.655 * (0.021)
**Butter and Oil**	−0.843 ** (0.001)	−0.882 ** (0.000)
**Eggs**	0.445 (0.147)	0.460 (0.132)
**Fish products**	0.549 (0.065)	0.454 (0.139)
**Sugar**	−0.823 ** (0.001)	−0.778 ** (0.003)
**Vegetable oil and other oils**	0.188 (0.558)	0.027 (0.935)

Note: ** Correlation is significant at the 0.01 level (2-tailed). * Correlation is significant at the 0.05 level (2-tailed).

## Data Availability

Data is contained within the article.
